# Defective Secretion of Islet Hormones in Chromogranin-B Deficient Mice

**DOI:** 10.1371/journal.pone.0008936

**Published:** 2010-01-28

**Authors:** Stefanie Obermüller, Federico Calegari, Angus King, Anders Lindqvist, Ingmar Lundquist, Albert Salehi, Maura Francolini, Patrizia Rosa, Patrik Rorsman, Wieland B. Huttner, Sebastian Barg

**Affiliations:** 1 Department of Clinical Sciences-Malmö, Lund University, Malmö, Sweden; 2 Max Planck Institute of Molecular Cell Biology and Genetics, Dresden, Germany; 3 CNR Institute of Neuroscience, Department of Medical Pharmacology, University of Milan, Milan, Italy; 4 Department of Neurobiology, University of Heidelberg, Heidelberg, Germany; 5 Oxford Centre for Diabetes, Endocrinology and Metabolism (OCDEM), University of Oxford, Churchill Hospital, Oxford, United Kingdom; 6 Department of Medical Cell Biology, Uppsala University, Uppsala, Sweden; University of Bremen, Germany

## Abstract

Granins are major constituents of dense-core secretory granules in neuroendocrine cells, but their function is still a matter of debate. Work in cell lines has suggested that the most abundant and ubiquitously expressed granins, chromogranin A and B (CgA and CgB), are involved in granulogenesis and protein sorting. Here we report the generation and characterization of mice lacking chromogranin B (CgB-ko), which were viable and fertile. Unlike neuroendocrine tissues, pancreatic islets of these animals lacked compensatory changes in other granins and were therefore analyzed in detail. Stimulated secretion of insulin, glucagon and somatostatin was reduced in CgB-ko islets, in parallel with somewhat impaired glucose clearance and reduced insulin release, but normal insulin sensitivity *in vivo*. CgB-ko islets lacked specifically the rapid initial phase of stimulated secretion, had elevated basal insulin release, and stored and released twice as much proinsulin as wildtype (wt) islets. Stimulated release of glucagon and somatostatin was reduced as well. Surprisingly, biogenesis, morphology and function of insulin granules were normal, and no differences were found with regard to β-cell stimulus-secretion coupling. We conclude that CgB is not required for normal insulin granule biogenesis or maintenance in vivo, but is essential for adequate secretion of islet hormones. Consequentially CgB-ko animals display some, but not all, hallmarks of human type-2 diabetes. However, the molecular mechanisms underlying this defect remain to be determined.

## Introduction

Granins form a family of highly acidic proteins that are primarily found in the lumen of dense-core secretory granules of endocrine cells and neurons. The most abundant members of this family are chromogranin A (CgA), chromogranin B (CgB), and secretogranin II (SgII) which are characterized by i) highly hydrophilic, acidic primary amino acid sequences, ii) the presence of multiple dibasic sites as potential targets for proteolytic processing, iii) a multitude of post-translational modifications, and iv) the tendency to self-aggregate at low pH/high Ca^2+^ conditions typical of the lumen of the trans-Golgi network (TGN) and secretory granules[1], [2], [3], [4]. The precise function of chromogranins is still debated, but evidence points in four main directions.

First, both CgA and CgB have been implicated in granulogenesis [1], [5]. Down-regulation of CgA [6] and CgB [7] resulted in the loss of secretory granules in PC12 cells and overexpression induced granule biogenesis in non-secretory cells [6], [7], [8], suggesting that granin expression is essential and sufficient to the neuroendocrine phenotype. This view has been challenged by the findings that granules can form independently of CgA in PC12 [9] and mouse chromaffin cells [10], [11] and that in non-secretory cells exogenous granins accumulate in dense compartments that may resemble, but are not identical to, secretory granules [9], [12], [13].

Second, granins play a role in the sorting and packaging of neuropeptides in granules within the TGN [14], [15], [16]. For example, in the TGN SgIII binds both to the proposed sorting receptor carboxypeptidase E (CPE) [17] and CgA and mediates targeting of proopiomelanocortin-derived peptides to granules [18]. Similarly, targeting of CgB to granules depends both on its self-aggregation and binding to an unknown sorting receptor [19], which suggests that granins act as an assembly factor in the TGN that may recruit other proteins into the budding granule [16].

Third, granins may participate in the structural matrix of the granule lumen [20] and facilitate storage of transmitter molecules such as ATP and catecholamines [21], [22]. Granins have pH-buffering capability and bind and release large amounts of ATP, Ca^2+^ and catecholamines [3], [22]. Knockout of CgA leads to a reduction in the amount of catecholamine stored in chromaffin granules [23].

Fourth, granin-derived peptides are secreted during regulated exocytosis and might thus exert hormonal, autocrine, and paracrine activities [4], [24], [25], [26]. The perhaps best known example is pancreastatin, a CgA derived peptide with strong inhibitory action on insulin secretion [25]. Antisera against CgB have been reported to stimulate insulin release [27].

Here we report the generation of a mouse line that lacks CgB (CgB-ko). These mice were viable and fertile but exhibited defects in hormone secretion from pancreatic islets, which were paralleled by slight glucose intolerance. Surprisingly, synthesis and function of insulin granules were normal, which argues against a role of CgB in granulogenesis.

## Results

### Generation of CgB-ko Mice

**Figure 1:**
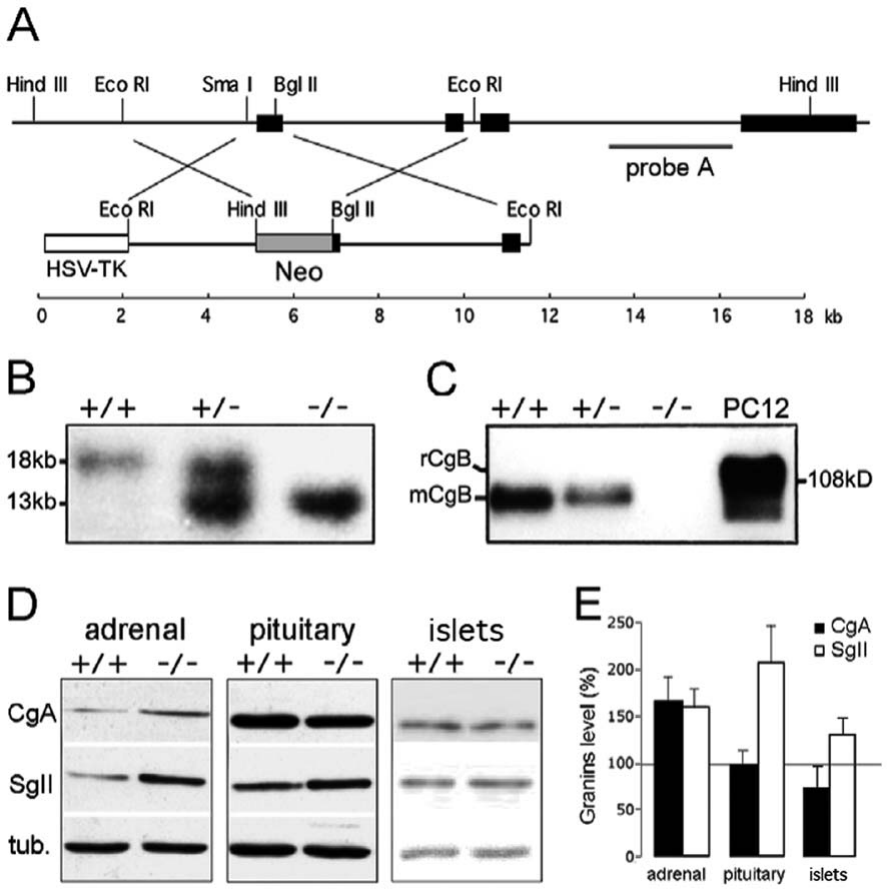
Generation of the CgB-ko mouse line. (**A**) Top, map of the *mCgb* locus (exons indicated as black bars) and region encoding the probe (probe A) used for Southern blot analysis. Bottom, construct used for homologous recombination containing the neomycin (Neo) and HSV-TK cassettes used for double drug selection. Relevant restriction sites and scale in kb are indicated. (**B**) Southern blot of HindIII-digested genomic DNA of CgB wt (+/+), heterozygous (+/-), and CgB-ko (−/−) mice. (**C**) Western blot for CgB in adrenals glands of wt (+/+), heterozygous (+/−) and CgB-ko (−/−) mice, and PC12 cells as control. Note the higher molecular weight of rat CgB (rCgB) from PC12 cells compared to that of mouse CgB (mCgB). (**D**) Western blot analysis of CgA and SgII expression, in adrenal gland, pituitary gland and pancreatic islets from wt (+/+) and CgB-ko (−/−) mice. Tubulin (tub) was used as loading control. (**E**) Quantification of experiments as in (E), for CgA (black bars) and SgII (white bars). Values represent expression relative to the corresponding wt sample (100%) after internal normalization with the tubulin signal.

The deletion of the mCgB gene was obtained by homologous recombination of a targeting vector which replaced 117 bases of the proximal promoter region and the first 29 bases of the coding sequence of CgB with the neomycin resistance gene (Fig 1A). ES clones were screened for homologous recombination by Southern blot analysis and positive clones were injected into C57BL/6J blastocysts to generate chimeras. CgB-ko mice were identified in the F2 by Southern blot analyses (Fig 1B) and lack of CgB protein confirmed by Western blot analysis of adrenal tissue (Fig 1C). Like CgA-ko mice [11], CgB-ko animals are viable and fertile, and showed no obvious phenotype with respect to weight, mating performance and overall behavior.

### Expression of Other Granins

The lack of an obvious phenotype in CgB-ko mice could be explained by compensatory overexpression of other granins such as CgA and SgII [11]. We therefore compared the abundance of CgA and SgII in adrenal, pituitary and isolated pancreatic islets obtained from 3–6 months old wt or CgB-ko mice, of both sexes, by Western blot analysis (Fig 1D). CgB-ko mice had i) increased levels of both CgA and SgII in adrenal glands (167±25% of wt, n = 8 and 163±20%, n = 12, respectively), ii) similar levels of CgA expression but a robust increase of SgII in pituitary glands (97±14%, n = 9 and 207±39%, n = 10, respectively), and iii) similar levels of both CgA and SgII expression in islets (73±22%, n = 4 and 131±17%, n = 4, respectively) (Fig 1D and E). Thus, the lack of CgB induces increased expression of other granins in some, but not all, endocrine tissues. Since the changes were minor in pancreatic islets, we decided to focus our study on the endocrine pancreas, where effects of CgB ablation can be studied in the absence of compensatory effects on other granins. Importantly, the expression of the granule membrane proteins carboxypeptidase E (CPE) and phogrin (IA-2β) was not altered in islets obtained from CgB-ko as compared to those obtained from wt mice (93±7%, n = 4 and 100±9%, n = 4; not shown), indicating that the overall constituents of secretory granules are similar in the absence of CgB.

### Decreased Hormone Secretion from Islets of Langerhans In Vitro

**Figure 2:**
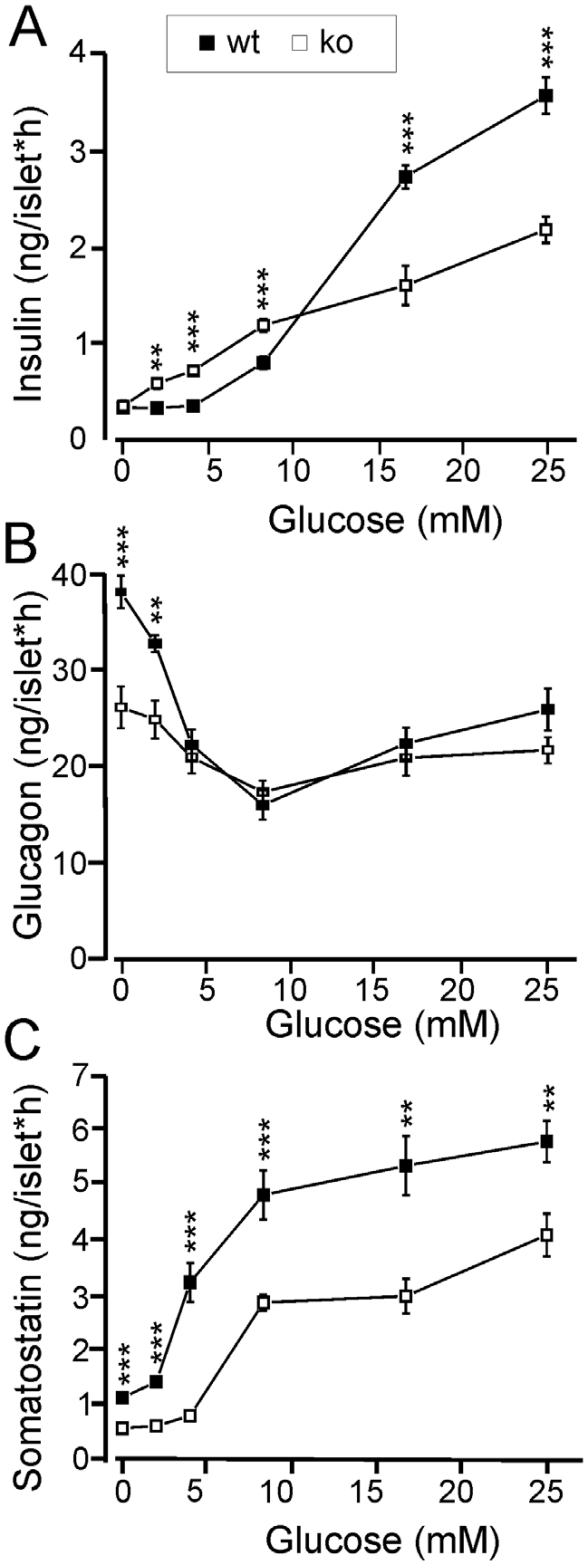
Hormone secretion from isolated pancreatic islets. Quantification of insulin (**A**), glucagon (**B**) and somatostatin (**C**) release from isolated wt (black squares) and CgB-ko (white squares) islets during 1 h incubation at various glucose concentrations (0–25 mM); n = 8. **P<0.01; ***P<0.001.

Islets were isolated and hormone release measured at glucose concentrations ranging from 0 to 25 mM (Fig 2). Insulin secretion from wt islets was observed at glucose >5 mM, and increased sigmoidally with the glucose concentration to a maximum rate of 3.6±0.5 ng/(islet*h) at 25 mM glucose. Half-maximal stimulation (EC_50_) was reached at 13 mM glucose (Fig 2A). Strikingly, islets from CgB-ko mice had a nearly linear response to glucose with a maximum release rate of only 2.2±0.4 ng/(islet*h) at 25 mM glucose. Insulin release at low glucose (2–8 mM) was significantly higher in CgB-ko than in wt islets, but still lower than in wt islets in stimulatory glucose (16–25 mM; p<0.01 at 2–25 mM glucose; see Fig 2A). Insulin content was similar in both groups, with 24±8 ng insulin/islet found in wt (29±2% of total protein, n = 9) and 34±9 ng/islet in the CgB-ko islets (34±4% of total protein, n = 8; data not shown).

Glucagon was measured in the same samples (Fig 2B). As expected, glucose had a bimodal effect on glucagon release with a maximal inhibition at 8 mM in both wt and CgB-ko islets. At 0–2 mM and again at 25 mM glucose, release in the CgB-ko was significantly reduced compared to wt. In the absence of glucose, CgB-ko islets released 26±6 pg/(islet^−1^*h), i.e. 30% less than wt islets which released 38±5 pg/(islet^−1^*h). Thus, in CgB-ko islets stimulated glucagon secretion at both low and high glucose concentrations was reduced compared to wt, while secretion at intermediate blood glucose (8 mM point) was unchanged.

Somatostatin was released from wt islets at a rate of 1.1±0.1 pmol/(islet*h) at 0 mM glucose, and increased sigmoidally with the glucose concentration (EC_50_ = 4.4 mM) to a maximum of 5.7±0.4 pmol/(islet*h) in 25 mM glucose (Fig 2C). Islets from CgB-ko mice showed a similar glucose dependent increase of somatostatin release (EC_50_ = 6.6 mM), but secretion was reduced to about half of that measured from wt islets, at all glucose concentrations (p<0.01) (Fig 2C).

### Time Course of Glucose-Dependent Insulin Release from Isolated Pancreatic Islets

**Figure 3:**
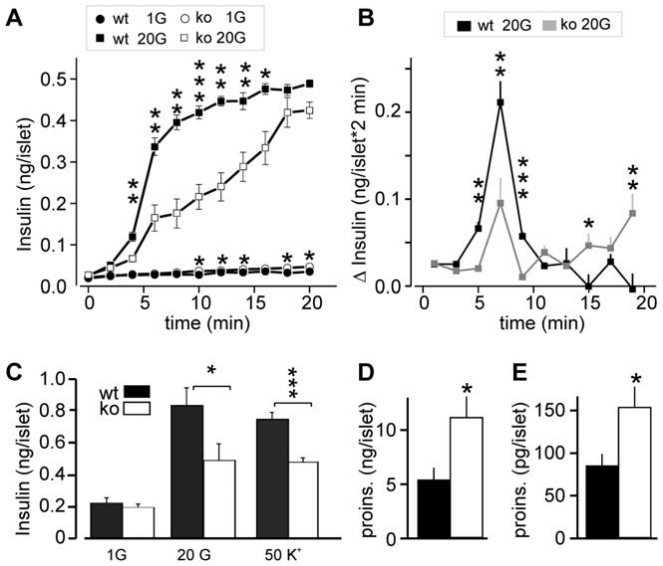
Timecourse of glucose- and K^+^-induced insulin release from isolated islets. (**A**) Isolated wt (black) and CgB-ko (white) islets were incubated in the presence of 1 mM (circles) or 20 mM (squares) glucose. Insulin in the medium was measured at 2 min intervals during 20 min after adjusting the glucose concentration; n = 4. (**B**) Same data as in A, but plotted as release per 2 min. (**C**) Insulin secretion from wt islets (black) and CgB-ko (white) islets was measured after 15 min incubation in the following conditions: 1 mM glucose+5 mM K^+^ (1G), 20 mM glucose+5 mM K^+^ (20 G) and 1 mM glucose+50 mM K^+^ (50 K^+^); n =  10. (**D**) Proinsulin content of wt (black) and CgB-ko islets (white); (n = 8 each). (**E**) Proinsulin release during 30 min batch incubations in buffer containing 20 mM glucose. *P<0.05; **P<0.01; ***P<0.001.

Next, we measured the time course of insulin secretion from isolated islets (Fig 3A-B). Similar to what is observed in perifusion experiments, wt islets responded to high glucose (20 mM) with a biphasic timecourse characterized by a transient rapid phase of release followed by a slower sustained phase. The peak rate 4–6 min after the onset of the glucose challenge amounted to 0.105±0.002 ng/(islet*min) in wt, but was reduced to a third in CgB-ko islets (maximum 0.034±0.004 ng/(islet*min); p<0.002) (Fig 3B). Release during the sustained phase (8–20 min) was slower in wt than in CgB-ko islets (0.007±0.002 vs. 0.022±0.002 ng/(islet*min), p<0.001) (Fig 3A–B). CgB-ko islets also showed slightly increased basal secretion, confirming the results with static incubations (Fig 2A).

Unlike glucose, depolarization by elevated extracellular K^+^ does not sustain insulin release beyond the initial rapid phase. After 15 min of stimulation with 50 mM K^+^, wt islets had released 0.71±0.05 ng/islet and CgB-ko islets only 0.45±0.03 ng/islet (Fig 3C). For comparison, 20 mM glucose released 0.83±0.11 ng/islet in wt and 0.49±0.1 ng/islet in the CgB-ko (Fig 3C).

Since the lack of CgB might affect insulin storage or proinsulin processing, we quantified proinsulin by enzyme-linked immunosorbent assay (ELISA). Proinsulin content of wt islets was 5.4±1.2 ng/islet, similar to previously reported values in mouse [28]. CgB-ko islets contained twice as much proinsulin, 11.2±2.0 ng/islet (Fig 3D). Likewise, proinsulin secretion from CgB-ko islets stimulated with 20 mM glucose was elevated, with 85±14 and 154±25 pg/islet*30 min in wt and CgB-ko, respectively (Fig 3E).

Taken together, ablation of CgB leads to modest increase in proinsulin storage and release and selectively decreases the rapid initial release of insulin, which likely corresponds to 1^st^ phase insulin secretion.

### CgB-ko Mice Exhibit Abnormal Insulin Secretion In Vivo

**Figure 4:**
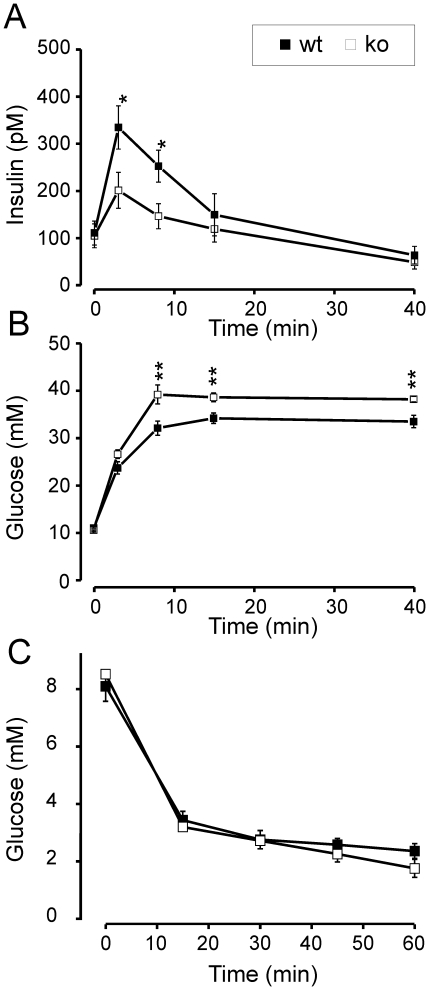
*In vivo* measurements of insulin secretion, glucose tolerance and insulin sensitivity. Time course of (**A**) plasma insulin concentration (pM), and (**B**) plasma glucose (mM) in response to i.p. glucose injection (3 g/kg) at time 0; wt (black squares, n = 12) and CgB-ko (white squares, n = 16). (**C**) plasma glucose (mM) in response to i.p. insulin injection (0.8U/kg). **P<0.01;*P<0.05.

We tested how the defects observed in islets might affect glucose tolerance of the mice. A bolus of glucose was injected intraperitoneally (i.p.), followed by analysis of blood samples (Fig 4). The initial plasma insulin concentration was similar in wt and CgB-ko mice (100±22 pM vs. 93±21 pM; Fig 4). All mice responded to the glucose challenge with a transient increase in plasma insulin, which then declined to baseline with a half-time of ∼10 min. The peak in plasma insulin was higher in the wt (332±46 pM), compared with CgB-ko (200±38 pM) (Fig 4A). Correspondingly, the wt mice were somewhat more resistant to the glucose challenge and plasma glucose reached peaks of 33.9±1.1 mM in wt and 38.3±0.9 mM in CgB-ko (Fig 4B). Lack of CgB might affect peripheral glucose uptake, as was recently shown for CgA [29]. We therefore tested whether insulin sensitivity was likewise affected in the CgB-ko animals. This was not the case since a single i.p. injection of insulin (0.8 U/kg body weight) caused near identical reductions in blood glucose over time in both wt and CgB-ko animals (Fig 4C).

### Granule Number and Morphology Is Unchanged in CgB-ko β-Cells

**Figure 5:**
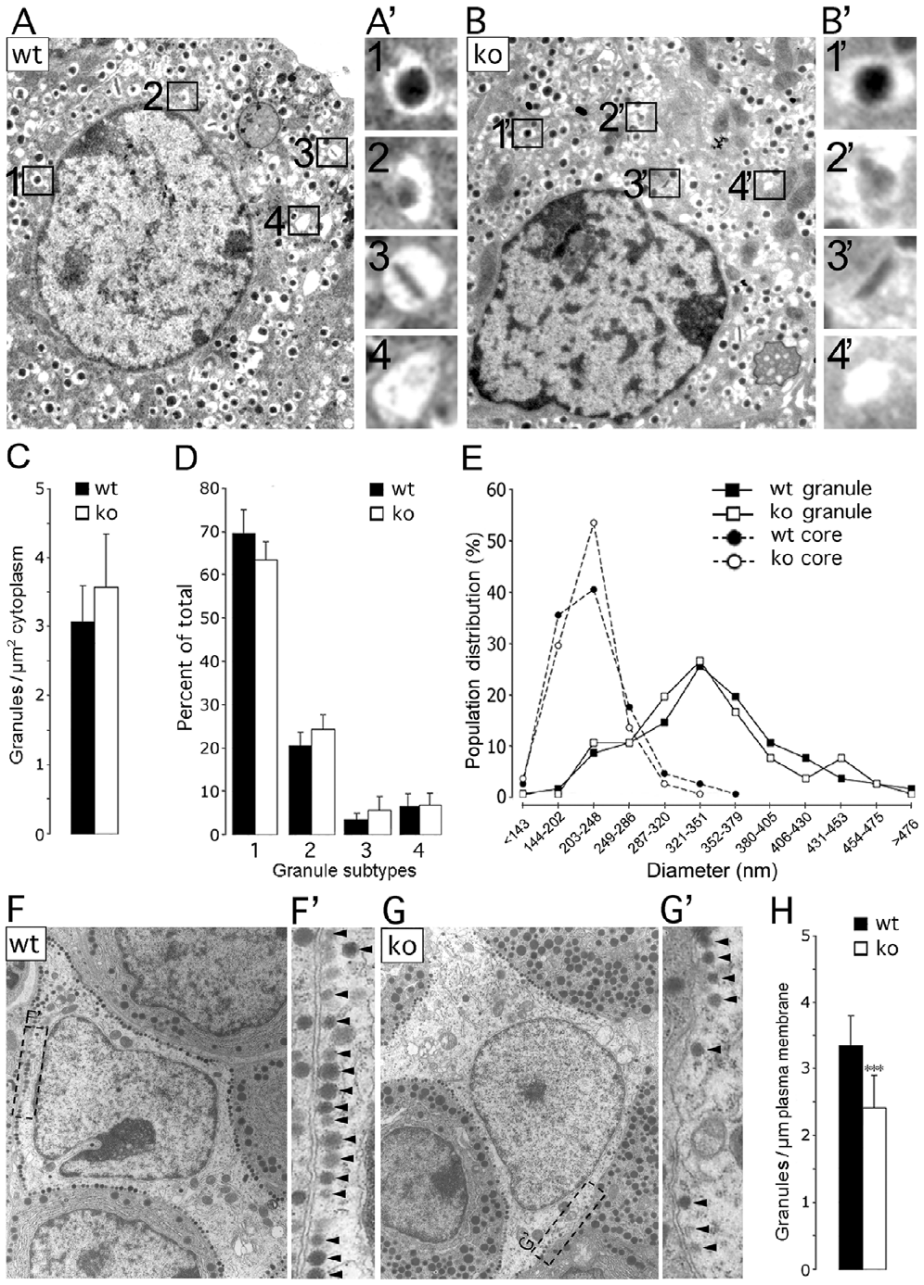
Ultrastructural analysis of insulin granules. (**A-B**) Electron micrographs of wt and CgB-ko β-cells. The insets (A' and B') illustrate magnified examples of the four granule subtypes: (1) mature, (2) immature, (3) crystal-containing and (4) empty. (**C-E**) Quantification and morphometric analysis of granules from wt (black bars and symbols) and CgB-ko (white bars and symbols) β-cells showing (C) number of granules per cytoplasm area, (D) abundance of the granule subtypes (see above), and (E) distribution of the area of mature granules (squares) and their dense-cores (circles). (**F-G**) Electron micrographs of adenocorticotropic cells from wt and CgB-ko mice. Insets A' and B' show magnified examples of granules (arrowheads) at the plasma membrane. (**H**) Quantification of density of granules along the plasma membrane of wt (black) and CgB-ko (white) mice.

Granins have been implicated in both biogenesis and maturation of secretory granules [1], [5], [6], [7], [8], [16]. We tested this by ultrastructure analysis of β-cells within isolated islets (Fig 5 A,B). Granule counts were similar in wt and CgB-ko with 3.1±0.6 (n = 9) and 3.5±0.8 (n = 9) granules/µm^2^ cytosol, respectively (Fig 5C). Granules were then classified according to their morphology, which reflects their maturation [30]. We assessed the relative abundance of 1) mature granules with electron-dense core, 2) immature granules with electron-translucent core, 3) granules with a crystal and, 4) empty granules (see examples in Fig 5A'-B'). The relative abundance of all types was similar in wt and CgB-ko, with most granules having a dense or opaque core (∼70%, Fig 5D). Wt cells had a slightly higher proportion of mature granules than CgB-ko (mean±sd: 74.3±4.5% and 68.0±4.4%, respectively; p<0.01), and correspondingly fewer immature granules (21.9±3.5% and 26.0±3.1%, respectively; p<0.02) (n = 9 cells wt and CgB-ko; 100–500 total granules counted each). Finally, the size of mature granules and their cores was analyzed. Histograms of the cross-section granule area in wt and CgB-ko essentially overlap (peaks around 0.09 µm^2^) (Fig 5E; squares), as do the histograms for the cross-section area of the electron-dense core (peaks around 0.03 µm^2^). The corresponding average value for the granule diameter (0.34 µm, assuming spherical geometry) is in good agreement with earlier measurements (357 nm) [31]. Therefore, lack of CgB has no major affect on insulin granule biogenesis or maturation, but causes a modest increase in immature granules at the expense of those that are mature. These changes are unlikely to be a primary cause of defected insulin secretion.

Some of the granules near the plasma membrane may be docked and therefore more likely to undergo exocytosis. However, the proportion of docked granules, defined as visually connected to the plasma membrane, was similar in wt and CgB-ko (9.3±3.5% and 9.8±3.3%, respectively). Moreover, no difference was observed in the proportion of granules found within 1.5 granule diameters of the plasma membrane of wt and CgB-ko β-cells (20±7.7% and 19.8±7.2%, respectively) (Fig 5F) (n = 9 cells for wt and CgB-ko; 100–500 total granules counted each).

Chromogranin dependent effects on granulogenesis have been observed in other tissues [5], [6], [7], [8], [9]. Since we failed to detect any morphological changes in CgB-ko insulin granules, we reasoned that such effects may be tissue specific. To test this, we repeated the ultrastructure analysis in pituitary adenocorticotropic cells (Fig 5G–E) and compared the density of granules in CgB wt and CgB-ko mice. Since most granules in these cells were near the plasma membrane, we analyzed the number of granules per µm plasma membrane. 30% fewer granules lined the plasma membrane of CgB-ko compared to wt cells (2.42±0.51 vs. 3.35±0.44 granules/µm, respectively; p<0.0001; n = 12 cells for wt and CgB-ko; 80–220 total granules counted each), which, together with the previous quantifications in wt and CgB-ko β-cells suggests a tissue-specific effect of CgB ablation on granulogenesis. No overall difference in either granule size or morphology was observed in adenocorticotropic cells (data not shown).

### Ca^2+^-Handling and the Exocytotic Machinery Are Unaffected by CgB Ablation

**Figure 6:**
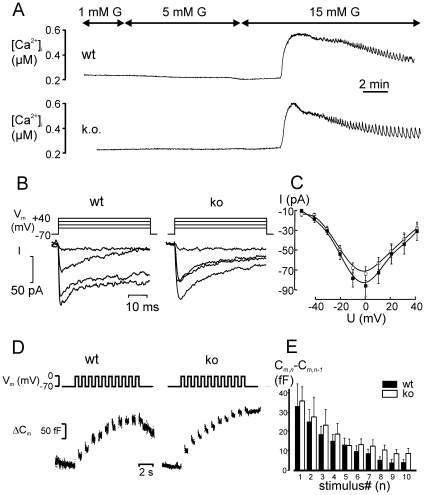
Stimulus-secretion coupling in isolated islets. (**A**) Measurements of [Ca^2+^]_i_ in intact wt (upper) and CgB-ko (lower) islets, representative of 25 and 27 experiments, respectively. The glucose concentration (G) was changed from 1 to 5 and later to 15 mM during the recording, as indicated. Note that the Ca^2+^-signal lags by >2 min, reflecting the time β-cell need to metabolize the sugar. (**B**) Whole-cell Ca^2+^-currents elicited by voltage-clamp depolarizations (50 ms from −70 mV to −40, −20, 0 and +20 mV) in β-cells on the periphery of intact isolated wt (left) and CgB-ko (right) islets, as indicated. (**C**) Current-voltage (IV) relationships in wt (black) CgB-ko (white) cells, from analysis of the peak currents in recordings as in B. (**D**) Whole-cell capacitance recordings (ΔC_m_, lower) elicited by trains of depolarizations from −70 to 0 mV (top) in β-cells on the periphery of isolated wt (left) and CgB-ko (right) islets. (**E**) Analysis of the increase in whole-cell capacitance evoked by each depolarization (ΔC_m_,_n_–ΔC_m_,_n−1_) and plotted against time; wt (black) and CgB-ko (white).

Insulin secretion is triggered by an increase in cytosolic calcium ([Ca^2+^]_i_) and altered Ca^2+^ handling could underlie the differences in the secretory capacity. We therefore measured ([Ca^2+^]_i_) in glucose stimulated islets using Fura-2 (Fig 6A). At 1 mM glucose, [Ca^2+^]_i_ averaged 104±14 nM (n = 27) in wt and 124.5±13 nM (n = 25) in CgB-ko islets. Increasing glucose to 5 mM did not change [Ca^2+^]_i_, while a further increase to 15 mM glucose resulted in a prominent, long-lasting increase in [Ca^2+^]_i_. Peak [Ca^2+^]_i_ was reached after ∼2 min and was higher than at baseline (1 mM), by 326±28 nM in wt and 349±25 nM in CgB-ko islets. [Ca^2+^]_i_ then slowly declined towards the pre-stimulatory level, with regular oscillations superimposed. These oscillations were similar in both groups (Fig 6A); they occurred with frequencies of 3±0.4 min^−1^ (wt) and 2.7±0.2 min^−1^ (CgB-ko) and had amplitudes of 113±10 nM (wt) and 128±12 nM (CgB-ko).

Voltage-dependent Ca^2+^-currents were measured by patch clamp. The Ca^2+^-current-voltage (IV)-relationships obtained from isolated β-cells of wt and CgB-ko mice were essentially identical, with maximal peak currents of 74±7 pA (wt, n = 8) and 86±10 pA (CgB-ko, n = 8), both elicited by depolarizations to 0 mV (Fig 6B-C). Half-maximal activation (V_half_) was at -22 mV for both groups with slopes of 6.6 and 7.8 mV/decade in wt and CgB-ko, respectively.

Ablation of CgB may affect the exocytotic machinery directly, which we tested by capacitance measurements of exocytosis in β-cells within isolated islets. Exocytosis was elicited by a train (1 s^−1^) of ten voltage-clamp depolarizations from −70 to 0 mV, each lasting 500 ms (Fig 6D). Ca^2+^-influx during the depolarizations was similar in both groups (not shown). The first depolarization elicited a capacitance increase of 33±11 fF in wt and 36±7 fF in CgB-ko islets. Subsequent responses gradually declined to 4±1 fF in wt and were, if anything, slightly higher in CgB-ko islets with 9±2 fF (Fig 6E). Thus, CgB is not required for proper function of the exocytotic machinery.

### Granule Stability during Individual Exocytotic Events

**Figure 7:**
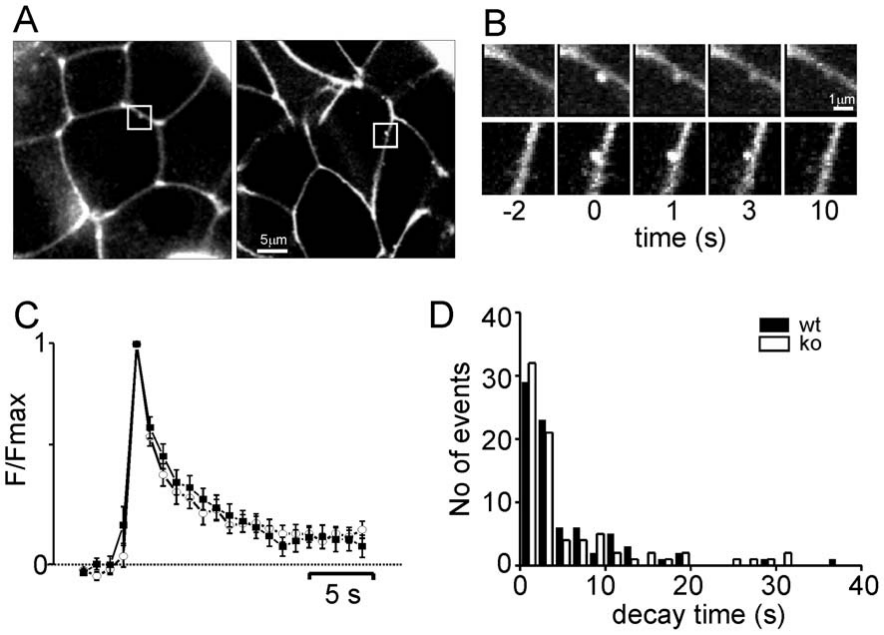
Kinetics of nucleotide release and granule integrity during exocytosis. (**A**) 2-photon confocal images of isolated islets bathed in sulforhodamine-B; images were acquired with a frequency of 1 s^−1^. The squares highlight fluorescent spots that appeared in response to a 20 mM glucose challenge in wt (left) and CgB-ko (right) islets. (**B**) Enlarged image sequences of the fluorescent transients highlighted in C; wt (upper row) and CgB-ko (lower row) islets. Times, in seconds, are relative to the appearance of the transient. (**C**) Averages of peak-aligned intensity plots, and (**D**) distribution of the time constants of mono-exponential functions (τ) fitted to the decay phase of events as in D; wt (black, n = 9 islets) and CgB-ko (white, n = 10 islets).

To study the structural integrity of individual insulin granules during exocytosis, we imaged uptake of the fluid-phase tracer sulforhodamine-B (SRB). This dye rapidly diffuses through the open fusion pore of granules and labels their lumen until the subsequent collapse of the granule ghost [32]. Isolated islets were pre-incubated in 5 mM glucose in presence of SRB and then challenged with 20 mM glucose in the continued presence of the dye. Shortly after increasing the glucose concentration transient bright spots were observed at the periphery of individual cells (Fig 7C-D). The frequency of such events in the imaged area (1,340 µm^2^) was 0.04±0.03 min^−1^ in 5 mM glucose and increased to 1.7±0.4 min^−1^ in 20 mM glucose (12 wt islets from 3 mice). Diazoxide (200 µM) prevented their occurrence (0.08±0.08 min^−1^ in 20 mM glucose). Similar results were obtained in CgB-ko islets, with frequencies of 0.05±0.03 s^−1^ in 5 mM glucose and 1.6±0.03 s^−1^ in 20 mM (n = 16). For most events the fluorescence increased instantaneously and then decayed, 81% of events in wt and 77% in CgB-ko reached baseline within 10 s (Fig 7E-F). There was no significant difference in the decay times (6.8±1.8 s in wt and 10.4±2.1 s in CgB-ko; medians 2.8 and 3.0 s). Thus, CgB ablation does not affect the stability of granules during glucose-induced exocytosis, and the data confirm that exocytosis proceeded at similar rates in wt and CgB-ko.

## Discussion

We have generated a mouse line deficient in CgB and studied the effects of this mutation at the molecular, cellular and systemic level. The analysis was focused on pancreatic islets, where compensatory upregulation of other granins was relatively minor. The study leads to two main conclusions, which are discussed here: 1) lack of CgB does not affect biosynthesis or function of insulin granules, and 2) islet hormone secretion is generally decreased in the absence of CgB (except at low glucose; Fig 2A). However, the mechanisms behind the altered hormone secretion, in particular the paradox between decreased hormone release and normal exocytosis, remain to be explained.

### CgB-Deficiency Affects Insulin Release but Not Exocytosis

Islets from CgB-ko mice had defects in the secretion of insulin, glucagon and somatostatin. Specifically, they lacked the rapid initial phase of insulin secretion, while basal insulin release was elevated. Despite disturbed insulin secretion, the mice appeared generally healthy with only mild glucose intolerance and normal insulin sensitivity. We speculate that this is due to two reasons: First, secretion of insulin, glucagon and somatostatin were reduced in parallel (Fig 2). Since somatostatin inhibits insulin secretion and glucagon opposes the actions of insulin, the net effect on glucose homeostasis is expected to be mild. Interestingly, the reduced inhibition by somatostatin may partially compensate for the insulin secretion defect, at least in stimulating conditions. Second, insulin secretion was actually elevated at low glucose concentrations and at later timepoints during glucose stimulation, which may lead to improved glucose clearance. The cause for this hypersecretion is not known, but it is unlikely to be related to somatostatin since it plays only a minor role at low glucose [33].

Paradoxically, the exocytotic machinery itself was normal and an equal number of granules underwent exocytosis upon stimulation. Both depolarization-evoked capacitance increases (Fig 6) and the frequencies of glucose-stimulated exocytosis events (Fig 7) were similar in wt and CgB-ko islets. How can insulin secretion be different when rates of exocytosis are not? We acknowledge that the conditions of the release assay (Fig 2a) and the capacitance measurements are not necessarily comparable (Fig 6). For example, including cAMP in the patch pipette may have overridden differences that normally exist in the islets, and the release assays were performed as batch incubations where secretory products accumulate. However, assuming that the rate of exocytosis is unchanged in CgB-ko in vivo, as suggested by the islet SRB recordings (Fig 7), a reasonable conclusion is that less insulin was released from each exocytosing granule.

In turn, this would imply that there is either less insulin stored per granule or that insulin is somehow retained by the granule membrane during exocytosis. The rate of insulin release from primary β-cell granules depends on the dispersion of the granular insulin condensate [34] and it is conceivable that the stability of this condensate is affected by loss of CgB. Indeed, CgB participates in the formation of the granular matrix [3], [26], [35] and interacts with peptide cargo [16]. This is relevant since insulin granules are capable of kiss-and-run type exocytosis [36] and retain their peptide cargo during such events [37], [38]. In contrast, exocytosis events where the fusion pore opens sufficiently to allow insulin release are mostly irreversible [32], [39], [40]. We used 2-photon imaging of SRB uptake to indirectly estimate the proportion of the two exocytosis modes. Kiss-and-run exocytosis allows SRB to enter the lumen, and the now labeled granule remains intact and visible *after* exocytosis. The mechanism should therefore result in longer average event lifetimes in the SRB assay, compared with full fusion (Fig 7). Since the lifetimes were unchanged in the CgB-ko islets (Fig 7F), it is unlikely that increased kiss-and-run can explain the decreased insulin secretion.

### Sorting and Processing of Proinsulin

Alternatively, the insulin content could be decreased due to impaired proinsulin processing, at least in a subset of granules. Indeed, the CgB-ko stored and released more proinsulin. Since the conversion of proinsulin to insulin occurs within the granules, this may suggest that proinsulin is either missorted into the constitutive pathway or poorly converted to mature insulin. Sorting into nascent granules is facilitated by the self-aggregation of cargo proteins in the TGN lumen [8], [41], [42]. Proinsulin does not self-aggregate appreciably and may therefore depend on additional factors, such as granins, for correct sorting to granules [16], [43]. However, the content of mature insulin in wt and CgB-ko islets was similar; even in the CgB-ko most of the synthesized insulin reached the regulated secretory pathway. CgB is therefore not a rate-limiting factor for proinsulin sorting. Conceivably, lack of CgB could also slow down the rate at which proinsulin is converted to mature insulin, as is the case in islets of human type-2 diabetics [44]. Newer granules would then contain more proinsulin, which does not crystallize within the granule [45], and therefore appear as immature in electron micrographs. Indeed, we found more immature granules (+19%) and correspondingly fewer mature granules (−8%) in CgB-ko β-cells. Since younger granules are preferentially released [46], [47], this scenario may explain both the increase in stored and released proinsulin and in the CgB-ko and why insulin secretion is impaired only during the first few minutes after glucose stimulation.

### Secretory Granule Biogenesis

Granins have been proposed as granulogenic factors because they can induce granular structures when expressed exogenously in non-secretory cells and because the number of granules in neuroendocrine cells depends on their expression levels [6], [7]. In the present study we found no difference in granule number or morphology in pancreatic islets of CgB-ko mice (Fig 5). Furthermore, expression of the granule proteins CPE and phogrin was unchanged (not shown) and regulated exocytosis of insulin granules was normal. Thus, CgB is not required for the formation of functional insulin granules. In contrast, CgB-ko adenocorticotropic cells had ∼30% few granules than wt, suggesting that the requirement for CgB for granulogenesis is cell- or tissue-specific. This may explain at least part of the current controversy whether granins induce granule formation, or not [5], [6], [7], [8], [9]. It is possible that some of these differences relate to the relative amounts of CgB and other granins (especially CgA), which may have similar granulogenic function and thus compensate for the lack of CgB. In this scenario, granulogenesis may not be affected in cells that normally contain relatively small amounts of CgB since it is compensated for by the presence of other, more abundant granins. In contrast to granulogenesis, we observed changes in hormone secretion in the CgB-ko that were actually more pronounced than in a CgA-ko [48]. Evidently, these changes are not compensated for by other granins.

### CgB and Human Disease

CgB-deficiency in mice leads to a phenotype with some hallmarks of human type-2 diabetes including loss of the initial rapid phase of insulin secretion and hypersecretion of proinsulin. Polymorphisms in either the CgB gene or factors affecting its expression could thus predispose to human diabetes. Such polymorphisms have already been linked to hypertension [49] and schizophrenia [50], a disease that correlates with increased risk for type-2 diabetes. Moreover, ablation of the forkhead transcription factor Foxa2 in mice induced hyperinsulinemic hypoglycemia in parallel with a 3-fold increase in CgB mRNA [51]. Clearly, further studies are required to understand the mechanism by which CgB affects secretion and whether the protein plays any role in diabetes. Such studies may be facilitated by the CgB-ko mouse model described here.

## Materials and Methods

### Ethics Statement

Animal experiments in Lund were approved by the local ethics committee (Malmö/Lunds djurförsöksetiska nämnd at Lunds tingsrätt) and conformed to Swedish animal protection laws and applicable guidelines (djurskyddslagen 1988:534; djurskyddsförordningen 1988:539; djurskyddsmyndigheten DFS 2004:4). Those in Germany were approved by the Regierungspräsidium Dresden, Germany (license number: 24D-9168.24-9-2005-3) and conformed to German law.

### Generation of CgB-ko Mice

For details on the generation of the CgB-ko line see [52]. Briefly, the targeting construct used for homologous recombination in E14.1-129/Ola ES cells included the *H. simplex* virus thymidine kinase gene, 2.8 kb of mCgB proximal promoter region, the neomycin resistance gene, and the full genomic sequence, BglII-EcoRI excised, of intron 1 and exon 2 of mCghb corresponding to 3.6 kb (Fig 1A). ES cells were cultured in presence of 2 mM gancyclovir (Syntex Pharmaceuticals) and 300 mg/ml neomycin (Gibco). After double selection, clones were screened by Southern blot analysis of HindIII digested genomic DNA using 1.6 kb fragment from intron 1 of mCgb as probe. Five positive clones were injected into C57/Bl-6 blastocysts and four generated chimeras showed germline transmission. Subsequent generations were obtained by intercrossing and outcrossing with C57BL/6J mice and genotyped by Southern blot analysis. Finally, lines of wt and CgB-ko mice were established from 12 CgB^+/−^ founders, with >95% C57BL/6J genetic background. Generation of the two lines involved 5 generations of littermate inbreeding to homogenate their genetic background. Age and sex-matched pairs were used.

### Islet and β-cell Preparation

Animal experiments were approved by local authorities of the University of Lund, Sweden; University of Heidelberg, Germany; University of Milan, Italy and by the Regierungspräsidium Dresden, Germany. Mice were killed by cervical dislocation, the pancreas quickly excised and islets isolated by standard collagenase P (Sigma-Aldrich, Stockholm, Sweden) digestion. The islets were maintained in short-term tissue culture (<24 h) in RPMI-1640 supplemented with 10% calf serum, 100 U/ml penicillin and 10 µg/ml streptomycin (all from Life Technologies, Täby, Sweden).

### Electron Microscopy

Pancreatic islets were fixed in 2.5% glutaraldehyde for 1 h, treated with 1% osmium tetroxide, dehydrated and embedded in Durcupan (Sigma-Aldrich). Freshly dissected pituitaries were fixed in 4% paraformaldehyde, 0.2% glutaraldehyde in 0.12 M cacodylate buffer (pH 7.4, 2 h on ice), postfixed in 1% osmiumtetroxide (1 h) and embedded in EMBed-812 (Science Services). Samples were then sectioned (60–80 nm), mounted on Cu-grids and contrasted with uranyl acetate and lead citrate and examined by electron microscopy (Morgagni, FEI, Eindhoven, Netherlands or JEM 1230, Jeol-USA, Peabody, MA).

### Hormone Release Measurements

Release from isolated islets was carried out at 37°C in Krebs-Ringer bicarbonate buffer (KRB; pH 7.4) containing additional 0.1% BSA, 1 mM glucose, 10 mM HEPES and gassed with 95% O_2_ and 5% CO_2_. After preincubation for 30 min, the medium was replaced with KRB with the indicated concentrations of glucose or KCl (50 mM, equimolarly replacing NaCl). All batches contained 12 islets in 1 ml solution, except in Fig 3A,B (50 islets in 1.5 ml). For *in vivo* measurements, glucose was injected intraperitoneally (3 g/kg body weight, in 0.9% NaCl) and blood was sampled every 2 min [53]. Insulin was quantified by radioimmunoassay [54], using insulin(I-125) as tracer and an antibody against mouse-insulin (LR-9011 and LR1013K, Linco, Tyresö, Sweden). Glucagon, somatostatin and proinsulin were analyzed with a commercially available radio-immuno assay kits (RB 310 and RB306, Euro-Diagnostica, Malmö, Sweden) or ELISA (Rat Proinsulin 10-1185-01, Mercodia, Sweden). Islet insulin content was determined following acidic alcohol extraction. Plasma glucose concentrations were determined enzymatically. Insulin for in vivo testing was from NovoNordisk, Bagsvaerd, Denmark (NovoRapid).

### Measurements of Intracellular Calcium

[Ca^2+^]_i_ was recorded by ratiometric microfluorimetry [31]. Islets were pre-loaded with 3 µM fura-2-acetomethoxyester (Molecular Probes) and imaged at >510 nm using a 63x/1.25 objective (Carl Zeiss) and alternating excitation light (350/380 nm, 10 Hz) in a buffer containing (in mM) 140 NaCl, 3.6 KCl, 2 NaHCO_3_, 0.5 NaH_2_PO_4_, 0.5 MgSO_4_, 5 HEPES (pH 7.4), 2.5 CaCl_2_ and glucose as indicated, at 32°C. We focused several cell diameters into the islets to decrease the influence of peripheral non-β-cells. [Ca^2+^]_i_ was calculated offline using the equation [Ca^2+^]_i_  =  
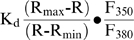
 assuming a *K*_d_ of 224 nM. *R*_max_ was estimated after addition of 60 µM ionomycin and background after quenching the fluorescence with 1 mM MnCl_2_.

### Electrophysiology

Measurements of exocytosis and calcium currents were performed in the whole-cell configuration using an EPC-10 amplifier (HEKA Elektronik). The intracellular solution consisted of (in mM) 125 CsCl, 10 NaCl, 1 MgCl_2_, 3 ATP-Mg, 0.1 cAMP, 0.05 EGTA and 5 HEPES (pH 7.15). The extracellular solution (EC) was (in mM) 138 NaCl, 5.6 KCl, 1.2 MgCl_2_, 2.6 CaCl_2_, 5 D-glucose, and 5 mM HEPES (pH 7.40) and held at 32°C. Capacitance was measured using 500 Hz, 20 mV sine waves. β-cells were identified by their electrophysiological properties[55].

### Rhodamine Uptake and Image Analysis

Islets were held with a suction pipette and incubated in EC with 1 mM sulforhodamine B (SRB) for 5 min. Exocytosis was then stimulated by adjusting glucose to 20 mM. SRB fluorescence was imaged at 1 Hz with 2-photon confocal microscopy (LSM510-Meta, Carl Zeiss) with a 40× objective (1.2 C-Apochromat, Carl Zeiss). SRB was excited at 800 nm and fluorescence detected at <560 nm. Exocytosis was detected manually in records obtained ∼2-15 min after solution change and quantified in circular regions (0.5 µm diameter) centered on the event location. The pre-stimulatory value was subtracted as local background.

### Statistics

Results are expressed as mean±S.E.M, except in Figs 1+5 where mean±S.D. is shown. Error bars smaller than the symbols are omitted. Significance of differences was assessed using Student's t-test.
